# Iron Status and Associated Malaria Risk Among African Children

**DOI:** 10.1093/cid/ciy791

**Published:** 2018-09-14

**Authors:** John Muthii Muriuki, Alexander J Mentzer, Wandia Kimita, Francis M Ndungu, Alex W Macharia, Emily L Webb, Swaib A Lule, Alireza Morovat, Adrian V S Hill, Philip Bejon, Alison M Elliott, Thomas N Williams, Sarah H Atkinson

**Affiliations:** 1 KEMRI-Wellcome Trust Research Programme, Kilifi, Kenya; 2 Wellcome Centre for Human Genetics, Nuffield Department of Medicine, University of Oxford; 3 London School of Hygiene and Tropical Medicine, United Kingdom; 4 Medical Research Council/Uganda Virus Research Institute and London School of Hygiene and Tropical Medicine Uganda Research Unit, Entebbe, Uganda; 5 Department of Clinical Biochemistry, Oxford University Hospitals; 6 Centre for Clinical Vaccinology and Tropical Medicine and the Jenner Institute Laboratories, University of Oxford; 7 Centre for Tropical Medicine and Global Health, Nuffield Department of Medicine, University of Oxford; 8 Department of Medicine, Imperial College, London; 9 Department of Paediatrics, University of Oxford, United Kingdom

**Keywords:** iron status, iron deficiency, malaria risk, African children

## Abstract

**Background:**

It remains unclear whether improving iron status increases malaria risk, and few studies have looked at the effect of host iron status on subsequent malaria infection. We therefore aimed to determine whether a child’s iron status influences their subsequent risk of malaria infection in sub-Saharan Africa.

**Methods:**

We assayed iron and inflammatory biomarkers from community-based cohorts of 1309 Kenyan and 1374 Ugandan children aged 0–7 years and conducted prospective surveillance for episodes of malaria. Poisson regression models were fitted to determine the effect of iron status on the incidence rate ratio (IRR) of malaria using longitudinal data covering a period of 6 months. Models were adjusted for age, sex, parasitemia, inflammation, and study site.

**Results:**

At baseline, the prevalence of iron deficiency (ID) was 36.9% and 34.6% in Kenyan and Ugandan children, respectively. ID anemia (IDA) affected 23.6% of Kenyan and 17.6% of Ugandan children. Malaria risk was lower in children with ID (IRR, 0.7; 95% confidence interval [CI], 0.6, 0.8; *P* < .001) and IDA (IRR, 0.7; 95% CI, 0.6, 0.9; *P* = .006). Low transferrin saturation (<10%) was similarly associated with lower malaria risk (IRR, 0.8; 95% CI, 0.6, 0.9; *P* = .016). However, variation in hepcidin, soluble transferrin receptors (sTfR), and hemoglobin/anemia was not associated with altered malaria risk.

**Conclusions:**

ID appears to protect against malaria infection in African children when defined using ferritin and transferrin saturation, but not when defined by hepcidin, sTfR, or hemoglobin. Additional research is required to determine causality.

**Clinical Trials Registration:**

ISRCTN32849447

## Teaser

Decreased ferritin and transferrin saturation are associated with protection against malaria in African children. Hepcidin, soluble transferrin receptor, and hemoglobin concentrations are not associated with malaria protection. These findings may reflect differences in parasite iron acquisition.

Iron deficiency (ID) and malaria remain important public health problems in African children [1, 2]. ID, the most common nutrient deficiency in preschool African children [3], is associated with poor brain development and long-term behavioral and cognitive impairments [4]. Similarly, malaria has devastating health effects in African children. In 2015, malaria caused an estimated 292000 deaths in African children aged <5 years [2] and remains a persistent and widespread problem in Africa, infecting 24% of the population at any one time [5].

The safety of iron supplementation has been a long-standing concern among policy makers and clinicians in malaria-endemic areas [6, 7]. In these areas, the World Health Organization (WHO) recommends iron supplementation in conjunction with effective malaria prevention and treatment strategies [8]. However, randomized, controlled trials of iron supplementation have reported conflicting findings [9, 10]. Furthermore, it is unclear whether iron supplementation might be unsafe because it improves iron status itself, thus resulting in a long-term increase in the risk of malaria.

Few observational studies have investigated the effect of iron status on malaria risk. These studies indicate that ID is associated with a reduced risk of both mild and severe *Plasmodium falciparum* malaria in African children [11–15] but have largely used ferritin-based definitions of ID. Little is known about whether other indicators of iron status (including hepcidin, hemoglobin, soluble transferrin receptors [sTfR], and transferrin saturation [TSAT]) influence malaria risk in humans. In mouse models, hepcidin has been shown to play a role in preventing superinfection by depriving the *Plasmodium* liver stage of iron [16], but studies in children have reported mixed findings [17, 18]. Two previous studies have reported that hemoglobin concentrations do not influence malaria risk [19, 20], while in vitro culture indicates otherwise [21]. There are no specific reports of the influence of sTfR and TSAT on malaria in humans.

In this study, our aim was to determine whether iron status influences the subsequent risk of malaria infection in 2683 Kenyan and Ugandan children, thus making this the largest observational study on iron status and risk of malaria to date with the most comprehensive range of iron markers.

## METHODS

### Ethical Approval

Ethical approval was provided by the Scientific Ethics Review Unit of the Kenya Medical Research Institute for the Kenyan cohort and the Uganda Virus Research Institute and the London School of Hygiene and Tropical Medicine for the Ugandan cohort.

### Study Population

This study used data from 2 African community-based cohorts of children in Kilifi, Kenya, and Entebbe, Uganda.

#### Kenya Cohort

The Kenya cohort included 3 community cohorts: Junju, Ngerenya, and malaria RTS,S vaccine. Junju and Ngerenya are ongoing rolling cohorts evaluating malaria immunity as described elsewhere [22]. The malaria RTS,S vaccine cohort was the RTS,S/AS01E vaccine trial against malaria that was conducted between 2007 and 2008 with continued active malaria surveillance for 8 years [23]. Within these cohorts, children are followed to a maximum age of 13 years with annual cross-sectional bleeds. The follow-ups involved weekly visits to assess for fever; if the temperature was above 37.5°C, a malaria blood film was taken. Iron biomarkers were measured from a single cross-sectional bleed based on the availability of plasma samples archived at −80°C.

#### Uganda Cohort

The Entebbe Mother and Baby Study is a prospective birth cohort study that was originally designed as a randomized, double-blind, placebo-controlled trial (ISRCTN32849447) to determine whether anti-helminthic treatment during pregnancy and early childhood was associated with differential responses to vaccination or incidence of infections such as pneumonia, diarrhea, or malaria [24]. Blood samples were collected at birth and at subsequent birthdays up to age 5 years. Markers of iron status were assayed from a single birthday based on the availability of stored samples. The study included longitudinal active surveillance of malaria and other infections during fortnightly home visits and quarterly clinic visits.

Longitudinal parasitemia data were obtained from active surveillance during the 6 months following measurement of iron biomarkers. For the study, 94% of Kenyan and 90% of Ugandan children were followed for 6 months, while the length of follow-up for the remainder ranged from 1 to 5 months. We chose a follow-up period of not more than 6 months since iron status may change over a longer follow-up period. Secondary analyses included a 1-year follow-up period. Clinical malaria data included microscopy-confirmed density of asexual *P. falciparum* parasitemia and temperature. Genotyping of hemoglobin types was conducted using polymerase chain reaction assay [25] with DNA extracted by Qiagen DNA Blood Mini Kit (Qiagen, West Sussex, United Kingdom).

### Measurement of Iron and Inflammatory Biomarkers

The assayed biomarkers of iron status included plasma ferritin (chemiluminescent microparticle immunoassay [CMI], Abbott Architect), hepcidin (DRG hepcidin 25 [bioactive] high sensitive enzyme-linked immunosorbent assay [ELISA] kit, DRG Diagnostics), sTfR (Human sTfR ELISA, BioVendor, CZ), iron (MULTIGENT iron calorimetric assay, Abbott Architect), transferrin (CMI, Abbott Architect), and hemoglobin (Medonic CA 530 hemoglobinometer). Since biomarkers of iron are influenced by inflammation, C-reactive protein (CRP) (MULTIGENT CRP Vario assay, Abbott Architect) was assayed to adjust for inflammation [26].

### Definitions

The following 2 definitions of ID were used: based on low ferritin defined as plasma ferritin <12 µg/L or <30 µg/L in the presence of inflammation (CRP >5 mg/L) in children aged <5 years or <15 µg/L in children aged ≥5 years [26], and TSAT <10% (calculated as iron in µmol/L/(transferrin in g/L × 25.1) × 100) [27]. TSAT was calculated in Kenya only because Ugandan plasma samples were stored in EDTA, which chelates iron. We did not define ID by hepcidin or sTfR since there are no internationally established cutoffs. Anemia was defined as hemoglobin <11 g/dL in children aged 0 to 4 years or hemoglobin <11.5 g/dL in children aged >4 years while ID anemia (IDA) was defined as low ferritin and anemia [28]. A malaria episode was defined as parasitemia and temperature >37.5°C. All malaria episodes that occurred during the follow-up period were included except those that occurred within 14 days of an initial presentation, which were regarded as recrudescence.

### Statistical Analyses

All analyses were conducted using STATA 13.0 (StataCorp, College Station, TX). Iron biomarkers (except hemoglobin) were log_e_-transformed to normalize their distributions. Geometric means of iron biomarkers and proportions of ID and anemia were computed. Two-tailed Student *t* tests were used to test for difference in means between groups. Poisson regression models of counts of malaria episodes were fitted as predicted by iron status (ID/anemia/individual iron biomarkers) and were adjusted for age, sex, parasitemia, inflammation, and study site. Difference in individual length of follow-up was accounted for in the model by including the length of follow-up as “exposure” in the model. We accounted for multiple episodes using robust cluster variance estimation, which takes into account correlations between multiple events. Secondary analyses involved excluding children with parasitemia or inflammation at baseline to mitigate the effects of concurrent infection on iron status [29]. We used Cox proportional hazards analyses to evaluate the temporal effect of iron status on malaria risk. A *P* value of <.05 was considered significant.

We searched the PubMed and Google Scholar databases with search terms that included “ID *or* ferritin *or* hepcidin *or* sTfR *or* TSAT *or* hemoglobin *or* anemia *and* malaria children.” We found 5 longitudinal studies that investigated the effect of ID on malaria risk. A metaanalysis of the current study and 4 previous longitudinal studies that reported risk ratios of the relationship between ID and malaria risk was performed using the “*metan*” command in STATA.

## RESULTS

### Baseline Characteristics of the Study Population

A total of 1309 Kenyan and 1374 Ugandan children aged 0–7 years and 1–5 years, respectively, were included in the analyses. Table 1 shows the characteristics of the study participants. At baseline, the prevalence of ID and IDA were 36.9% and 23.6% in Kenyan and 34.6% and 17.6% in Ugandan children, respectively. The prevalence of ID based on TSAT (measured in Kenya only) was 52.4%. The prevalence of malaria parasitemia was higher in Kenyan (20.1%) compared to Ugandan children (6.7%). During the 6-month follow-up, 31.1% of Kenyan and 14.3% of Ugandan children experienced at least 1 episode of malaria infection. Malaria incidence rate per child-year of follow-up was 0.6 in Kenya and 0.3 in Uganda.

**Table 1.** Baseline Characteristics of Study Participants

Characteristic	Kenya (n = 1309)	Uganda (n = 1374)
Mean age in years (range)	2.3 (0.0, 7.1)	2.3 (1.0, 5.1)
Males, n/total (%)	668/1309 (51.0)	696/1374 (51.7)
Malaria parasitemia, n/total (%)	261/1296 (20.1)	92/1371 (6.7)
Inflammation,^a^ n/total (%)	334/1264 (26.4)	316/1337 (23.6)
Iron deficiency,^b^ n/total (%)		
Low ferritin	457/1237 (36.9)	438/1267 (34.6)
TSAT < 10%	637/1215 (52.4)	n/a
Anemia,^c^ n/total (%)	526/765 (68.8)	533/1312 (40.6)
Iron deficiency anemia,^d^ n/total (%)	172/729 (23.6)	213/1209 (17.6)
Sickle cell trait, n (%)	157/1057 (14.9)	224/1355 (16.5)
Ferritin, n (geometric mean ± SD) in µg/L	1237 (20.8 ± 3.0)	1267 (20.8 ± 2.9)
Hepcidin, n (geometric mean ± SD) in µg/L	1202 (5.6 ± 3.6)	1333 (6.8 ± 3.3)
Soluble transferrin receptors, n (geometric mean ±SD) in mg/L	1296 (17.8 ± 1.5)	1343 (6.7 ± 2.0)
Hemoglobin, n (geometric mean ±SD) in g/dL	765 (10.1 ± 1.2)	1312 (11.0 ± 1.1)
TSAT, n (geometric mean ±SD) in %	1215 (9.3 ± 2.2)	n/a

Malaria incidence rate per child-year of follow-up was 0.6 in Kenya and 0.3 in Uganda.

Abbreviations: n/a, not available; SD, standard deviation; TSAT, transferrin saturation.

^a^Inflammation was defined as C-reactive protein >5 mg/L.

^b^Iron deficiency was defined using 2 definitions: low ferritin defined as plasma ferritin <12 µg/L or <30 µg/L in the presence of inflammation in children aged <5 years or <15 µg/L in children aged ≥5 years, and transferrin saturation <10% (available in 1215 Kenyan children only and not available (n/a) in Uganda).

^c^Anemia was defined as hemoglobin <11 g/dL in children aged 0 to 4 years or hemoglobin <11.5 g/dL in children aged >4 years. The range of hemoglobin was 5.1–14.7 in Kenya and 5.4–18.5 in Uganda. The interquartile range was 9.4–11.3 in Kenya and 10.3–12.1 in Uganda. Only 33 (1.6%) had severe anemia (Hb <7 g/dL in children aged <5 years or <8 g/dL in children aged >5 years).

^d^Iron deficiency anemia was defined as low ferritin and anemia.

### Higher Ferritin Concentrations and TSAT Are Positively Associated With Malaria Infection

**Figure 1.**
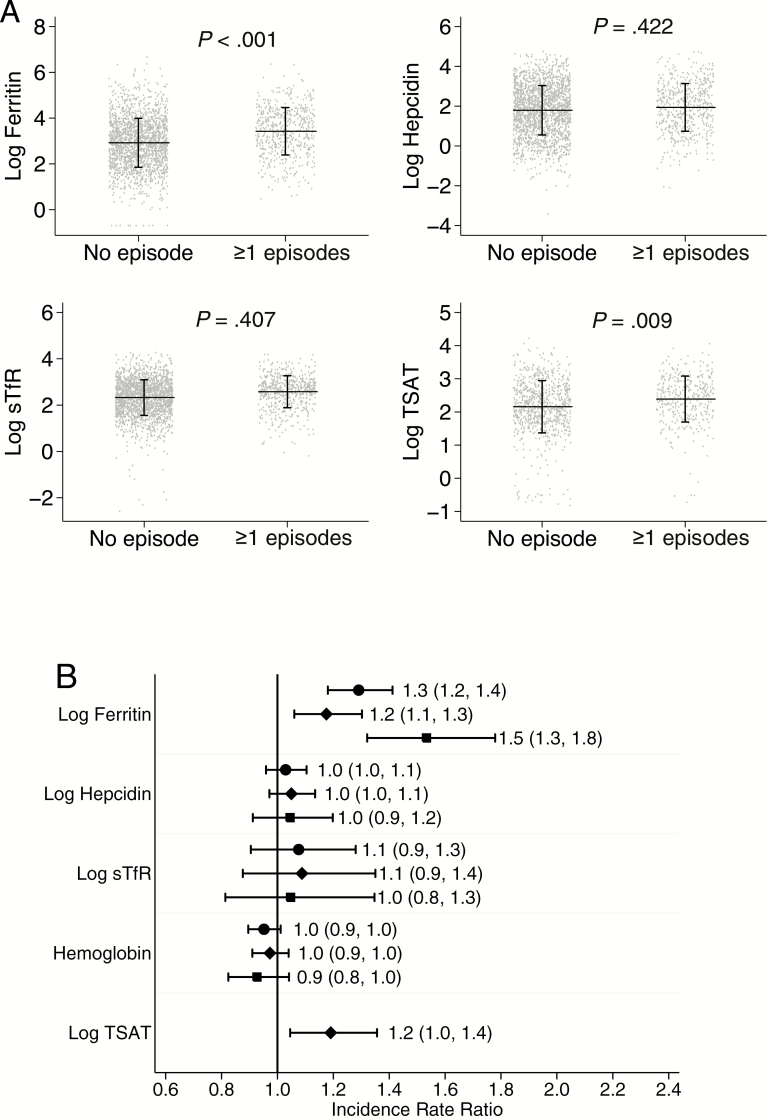
The effect of iron status on subsequent malaria. A, Scatter plots of iron biomarkers stratified by no subsequent malaria or one or more subsequent malaria episodes. Horizontal line indicates mean while vertical line indicates standard deviation. P value was derived from Poisson regression model. B, Adjusted incidence rate ratios for the effect of iron biomarkers on subsequent malaria episodes. Circle marker indicates overall, diamond Kenya and square Uganda. Labels indicate incidence rate ratio and 95% confidence intervals. Poisson regression models were adjusted for age, sex, parasitemia, inflammation, length of follow-up and study site. Maximum length of follow-up was 6 months. Abbreviations: sTfR, soluble transferrin receptor; TSAT, transferrin saturation.

Concentrations of ferritin and TSAT, but not other iron markers, were higher in children who subsequently developed a malaria episode (Figure 1A). Similarly, a unit increase in log ferritin was associated with an increased incidence rate ratio (IRR) for malaria overall (IRR, 1.3; 95% confidence interval [CI], 1.2, 1.4; *P* < .001) and in each cohort individually (Figure 1B). A unit increase in log TSAT was also associated with a 20% increased risk of malaria in Kenyan children (IRR, 1.2; 95% CI, 1.05, 1.4; *P* = .009). However, hepcidin, sTfR, and hemoglobin concentrations were not associated with subsequent risk of malaria (Figure 1B).

### ID Defined Using Ferritin or TSAT Protects Against Malaria Risk

ID defined by low ferritin concentrations and IDA were associated with a 30% reduction in the incidence of malaria infection (IRR, 0.7; 95% CI, 0.6, 0.8; *P* < .001 and IRR, 0.7; 95% CI, 0.6, 0.9; *P* = .006, respectively). These findings were consistent for the individual cohorts (Table 2). Likewise, ID defined by low TSAT reduced the risk of malaria in Kenyan children (IRR, 0.8; 95% CI, 0.6, 0.9; *P* = .016). However, anemia itself was not significantly associated with variation in malaria risk (Table 2).

**Table 2.** Incidence of Malaria by Iron Status and Anemia

	Kilifi, Kenya					Entebbe, Uganda					Overall				
Category	No.	No. of Episodes	Incidence	IRR (95% CI)	*P* Value	No.	No. of Episodes	Incidence	IRR (95% CI)	*P* Value	No.	No. of Episodes	Incidence	IRR (95% CI)	*P* Value
No ID (iron replete)	780	286	0.8	1	…	829	140	0.3	1	…	1609	426	0.6	1	…
ID (low ferritin)^a^	457	93	0.4	0.8 (0.6, 0.9)	.018	438	45	0.2	0.5 (0.4, 0.7)	<.001	895	138	0.3	0.7 (0.6, 0.8)	<.001
No ID (TSAT ≥10%)	578	224	0.8	1	…	n/a	n/a	n/a	n/a	n/a	578	224	0.8	1	…
ID (TSAT <10%)^b^	637	159	0.5	0.8 (0.6, 0.9)	.016	n/a	n/a	n/a	n/a	n/a	637	159	0.5	0.8 (0.6, 0.9)	.016
No anemia	239	97	0.8	1	…	779	93	0.2	1	…	1018	190	0.4	1	…
Anemia^c^	526	219	0.9	1.0 (0.8, 1.2)	.77	533	93	0.4	1.2 (0.9, 1.6)	.16	1059	312	0.6	1.1 (0.9, 1.3)	.17
No IDA	557	257	1.0	1	…	996	154	0.3	1	…	1553	411	0.5	1	…
IDA^d^	172	47	0.6	0.8 (0.6, 1.1)	.27	213	23	0.2	0.5 (0.3, 0.7)	<.001	385	70	0.4	0.7 (0.6, 0.9)	.006

Poisson regression models were adjusted for age, sex, parasitemia, inflammation, length of follow-up, and study site. Maximum length of follow-up was 6 months. Incidence defined as number of malaria episodes per child-year of follow-up. The number of episodes ranged from 0–5 in Kenya and 0–6 in Uganda; 101 Kenyan and 45 Ugandan children had multiple episodes.

Abbreviations: CI, confidence interval; ID, iron deficiency; IDA, iron deficiency anemia; IRR, incidence rate ratio; n/a, not available; TSAT, transferrin saturation.

^a^Iron deficiency (low ferritin) was defined as plasma ferritin <12 µg/L or <30 µg/L in the presence of inflammation (C-reactive protein >5 mg/L) in children aged <5 years or <15 µg/L in children aged ≥5 years otherwise iron replete.

^b^Transferrin saturation data were available in 1215 Kenyan children. Not available (n/a) for Uganda.

^c^Anemia was defined as hemoglobin <11 g/dL in children aged 0 to 4 years or hemoglobin <11.5 g/dL in children aged >4 years.

^d^Iron deficiency anemia was defined as low ferritin and anemia.

**Figure 2.**
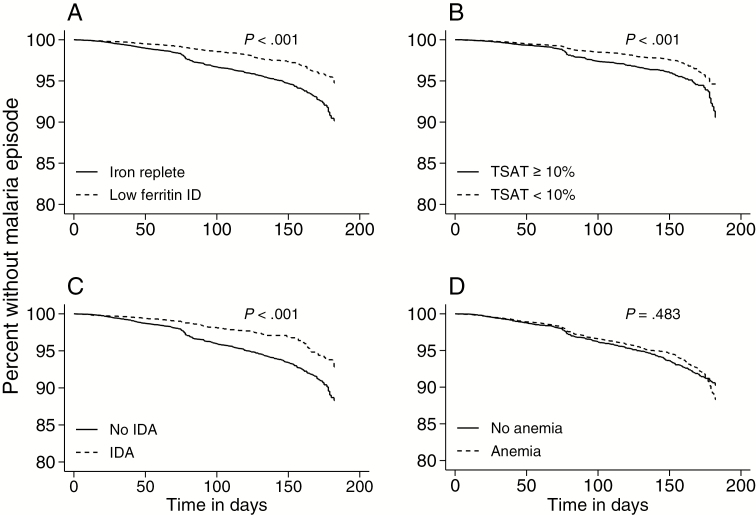
Kaplan-Meier curves of time to first malaria episode according to *(A*) iron deficiency (ID) defined by low ferritin, *(B*) ID defined by transferrin saturation (<10%), *(C *) ID anemia, and *(D*) anemia. *P* values were derived from log-rank tests for equality of survivor functions. Abbreviations: ID, iron deficiency; IDA, iron deficiency anemia; TSAT, transferrin saturation.

In Cox proportional hazards models, ID defined by low ferritin, ID defined by low TSAT, and IDA were associated with 40%, 20%, and 30% reduced risk of malaria, respectively (Figure 2A–C), for the 6 months of follow-up compared to iron-replete children. However, anemia was not associated with malaria risk (Figure 2D and Supplementary Table 1). We observed similar results regardless of whether the follow-up period was extended to 1 year, the children had malaria parasitemia or inflammation at baseline, age, or following adjustment for sickle cell trait (Supplementary Figures 1–5).

**Figure 3.**
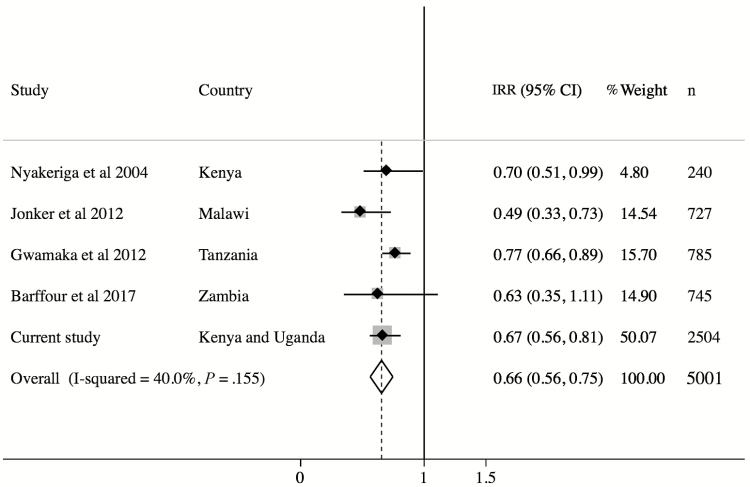
Metaanalysis of observational studies examining the relationship between iron deficiency (ID) and malaria risk. Study-specific estimates and their relative contribution (percentage weight and sample size) to overall estimates are shown. Definitions of ID varied by study: Nyakeriga et al 2004 [11], ferritin <12 µg/L plus transferrin saturation <10%; Jonker et al 2012 [12], ferritin <30 µg/L; Gwamaka et al 2012 [13], ferritin <30 µg/L if C-reactive protein (CRP) <8.2 mg/L or ferritin <70 µg/L if CRP >8.2 mg/L; Barffour et al 2017 [14], ferritin <12 µg/L in children aged <5 years or <15 µg/L in children aged ≥5 years; and current study, ferritin <12 µg/L or <30 µg/L if CRP >5 mg/L in children aged <5 years or <15 µg/L in children aged ≥5 years. Abbreviations: CI, confidence interval; IRR, incidence rate ratio.

A metaanalysis of observational studies examining the influence of ID on malaria risk is shown in Figure 3. All the studies report that ID, using a ferritin-based definition, protects against malaria infection, despite differences in study site, length of follow-up, and definition of ID (Supplementary Table 2). The overall estimate indicates that ID is associated with a 34% lower risk of malaria infection. We report the largest study to date.

## DISCUSSION

In this study, we report an observational analysis of the influence of iron status on subsequent malaria risk in 2683 Kenyan and Ugandan children. We found that ID, defined using either ferritin or TSAT, and IDA were associated with a lower risk of subsequent malaria infection. However, anemia (or hemoglobin concentrations), hepcidin, and sTfR were not significantly associated with variation in malaria risk.

Consistent with our findings, previous observational studies have reported that ID based on low ferritin concentrations confers protection against malaria infection in African children [11–15]. Nyakeriga et al reported a 30% reduction in clinical malaria during a 6-month follow-up of Kenyan children aged 8 months to 8 years [11]. Similarly, studies in Malawi (aged 6–60 months with 1 year of follow-up) [12] and Tanzania (birth to 3 years follow-up) [13] reported reduced malaria risk of 45% and 23%, respectively. Recently, a study in Zambian children aged 4–8 years followed for 6 months reported an increased risk of malaria in children with high ferritin concentrations [14]. These estimates are similar to our finding of a 30% reduction in malaria risk in iron-deficient children (0–7 years). The metaanalysis of these studies indicated that ID is associated with a 34% lower risk of malaria infection.

Also, we found that ID defined by TSAT was associated with a 20% reduction in the subsequent risk of malaria and that TSAT was positively associated with malaria incidence. Using a combination of low TSAT and low ferritin, Nyakeriga et al reported a 30% reduction in clinical malaria among iron-deficient children [11]. In support of our findings, *P. falciparum* has been demonstrated to obtain iron from transferrin using in vitro parasite culture [30]. Furthermore, Clark et al demonstrated that parasitized red blood cells utilize serum iron [31]. These studies indicate that increasing bioavailable transferrin-bound iron may predispose an individual to increased risk of malaria. Indeed, we show that higher TSAT may increase malaria risk in children.

So, how might ID protect against malaria infection? In in vitro parasite cultures, iron-deficient human erythrocytes are poorly infected by *P. falciparum* compared to those that are iron replete, and this protective effect is reversed by iron supplementation [32]. In mouse models, Matsuzaki-Moriya et al showed that during ID, macrophages cleared parasitized erythrocytes more efficiently, suggesting that either erythrocytes produced under iron-deficient conditions are easily phagocytized by macrophages or that there is an enhancement of macrophage function during ID [33]. Furthermore, ID has also been shown to upregulate nitric oxide, which has antiparasitic properties against *Plasmodium* [34]. Another possible explanation is that during ID, zinc is incorporated in place of iron during heme synthesis, leading to formation of zinc protoporphyrin that, in turn, is thought to inhibit formation of hemozoin (the parasite survival pigment) in a manner similar to quinolines [35].

We also report that anemia (or hemoglobin concentration) does not influence subsequent malaria risk. Similar observations from 2 previous studies have been reported. A recent longitudinal study in Papua New Guinean infants aged 3 months and followed for 1 year reported a nonsignificant association between lower hemoglobin concentrations and subsequent malaria infection [19]. Ghosh et al made a similar observation in Indian children [20]. Indeed, in both studies and in ours, it was found that anemic children have a nonsignificant increase in malaria risk rather than protection from malaria. In contrast, Goheen et al reported that anemia was associated with decreased in vitro growth rate of *P. falciparum* [21]. However, it is possible that in vitro parasite growth rate might not mimic direct malaria susceptibility for children. Moreover, hemoglobin has low sensitivity and specificity in determining body iron status due to the overlap of values in iron-deficient and iron-replete individuals [36] and the multiple overlapping causes of low hemoglobin concentrations in African children [37]. It remains unclear whether the malaria parasite utilizes heme iron in hemoglobin or has other sources and mechanisms of iron acquisition. There are suggestions that the parasite may utilize storage iron since bioavailable iron content increases in parasitized red blood cells as the parasite develops from ring stage to schizont [31].

We hypothesized that increased hepcidin concentrations may reduce malaria risk through sequestration of iron within macrophages and enterocytes [38], thereby starving liver-stage *Plasmodium* [16]. Since the parasite requires iron for growth, it has been suggested that withholding iron from hepatocytes inhibits the development of malaria [16]. Furthermore, high cord blood hepcidin has been associated with decreased risk of clinical malaria, although not parasitemia or severe malaria, in Tanzanian infants [18]. However, in agreement with our study in an independent cohort of Kenyan children [17], we found no association between hepcidin concentrations and clinical malaria episodes. Differences in age or environmental factors may account for the different findings. Moreover, the high prevalence of ID, which is normally associated with decreased hepcidin and reduced the risk of malaria in our participants, may counter a possible protective role of hepcidin.

Our data indicate that a child’s erythropoietic drive (as measured by sTfR) does not influence their subsequent risk of malaria infection. The expression of sTfR increases with both ID and expanded erythropoiesis (with the latter being more influential) [39], factors that might have opposing effects on malaria risk. For example, increased erythropoiesis may increase the risk of malaria since *Plasmodium* parasites preferentially infect young red blood cells [40], whereas ID may be protective. These opposing effects could explain why sTfR concentrations were not associated with malaria risk in our study. It is also known that malaria itself causes increased sTfR concentrations [39, 41].

A major challenge in this study is that iron biomarkers are influenced by infections and inflammatory processes that may confound the effect of iron status on malaria infection [29]. To mitigate the potential confounding effects of infection on iron biomarkers, we excluded children with inflammation or malaria parasitemia at the time of iron measurement in secondary analyses and observed similar results (Supplementary Figure 2). Additionally, other potential confounders such as age, sex, length of follow-up, and study site were adjusted for in regression models. Limitations of the study were lack of TSAT concentrations in Ugandan children and that only febrile malaria was included for the malaria episodes. Strengths of our study included its large size (n = 2683 children) across 2 study sites and that we used multiple iron biomarkers in order to determine their individual effects on malaria risk, making it the largest and most definitive observational study to address the question of iron status and risk of malaria infection.

Our findings, which are in agreement with other studies, suggest that ID protects children against malaria infection and thus that improving iron status may predispose African children to infection. Interestingly, of the iron biomarkers, only higher concentrations of ferritin and TSAT were predictive of increased rates of subsequent malaria, perhaps reflecting differences in their relationship to parasite mechanisms of iron acquisition. Although WHO recommends iron supplementation coupled with malaria treatment and prevention strategies in malaria-endemic areas [8], these strategies remain difficult to implement. Thus, it is important to establish whether improved iron status increases malaria risk since this would necessitate long-term malaria prevention and treatment programs. However, our findings and those from other studies do not necessarily imply causality since observational studies may be subject to confounding and reverse causation, for example, prior malaria exposure might lead to both ID (due to raised hepcidin concentrations blocking iron absorption) and the acquisition of protective immunity against malaria, while malaria itself increases ferritin levels. Since ID prevents children from reaching their developmental potential, it is important to establish causality in the iron–malaria relationship. Thus, these data warrant additional large-scale studies, including studies that utilize genetic variants associated with iron status to infer causality (Mendelian randomization) and prospective interventional trials.

## Supplementary Data

Supplementary materials are available at *Clinical Infectious Diseases* online. Consisting of data provided by the authors to benefit the reader, the posted materials are not copyedited and are the sole responsibility of the authors, so questions or comments should be addressed to the corresponding author.

ciy791_suppl_Supplementary_Material

## Notes

***Acknowledgments.*** We thank our colleagues Barnes Kitsao, Jennifer Musyoki, and Jedidah Mwacharo at the Kenya Medical Research Institute (KEMRI)-Wellcome Trust Research Programme for their support in retrieving archived samples. We also thank the team at the Entebbe Mother and Baby Study under the Medical Research Council (MRC)/Uganda Virus Research Institute and London School of Hygiene and Tropical Medicine Uganda Research Unit. This study is published with permission from the Director of KEMRI.

***Financial support.*** This work was funded by the Wellcome Trust (grant 110255/Z/15/Z to S. H. A., grant 202800/Z/16/Z to T. N. W., and grant 106289/Z/14/Z to A. J. M.) and the DELTAS Africa Initiative (DEL-15-003). The DELTAS Africa Initiative is an independent funding scheme of the Alliance for Accelerating Excellence in Science in Africa under the African Academy of Sciences and is supported by the New Partnership for Africa’s Development Planning and Coordinating Agency with funding from the Wellcome Trust (107769/Z/10/Z) and the UK government. The Ugandan cohort was supported by Wellcome Trust grants (064693, 079110, and 95778 to A. M. E.) with additional support from the Medical Research Council and Department for International Development, UK Government under the MRC/DfID concordat.

***Potential conflicts of interest.*** All authors: No reported conflicts of interest. All authors have submitted the ICMJE Form for Disclosure of Potential Conflicts of Interest. Conflicts that the editors consider relevant to the content of the manuscript have been disclosed.
