# Supplementary material for: Iron Status and Associated Malaria Risk Among African Children
Source: Clin Infect Dis. 2018 Sep 14;68(11):1807–14. doi: 10.1093/cid/ciy791 (PMC6522755; doi:10.1093/cid/ciy791)
Supplement: ciy791_suppl_Supplementary_Material [file ciy791_suppl_supplementary_material.docx]

**Supplementary Table 1. Cox proportional hazards regression models for predicting malaria risk**

|  | **Unadjusted**  **HR (95% CI)** | **P value** | **Adjusted^[[1]](#endnote-1)^**  **HR (95% CI)** | **P value** |
| --- | --- | --- | --- | --- |
| ID (low ferritin)^[[2]](#endnote-2)^ | 0.5 (0.4, 0.6) | <0.001 | 0.6 (0.5, 0.7) | <0.001 |
| ID (TSAT<10%) | 0.6 (0.5, 0.7) | <0.001 | 0.8 (0.6, 0.9) | 0.008 |
| Anemia^[[3]](#endnote-3)^ | 0.9 (0.8, 1.1) | 0.488 | 1.2 (0.9, 1.4) | 0.085 |
| Iron deficiency anemia^[[4]](#endnote-4)^ | 0.5 (0.4, 0.6) | <0.001 | 0.7 (0.6, 0.9) | 0.003 |

**Supplementary Table 2. A summary of observational studies examining the relationship between iron deficiency and malaria risk**

| **Study** | **Country** | **Age (months)** | **Sample size** | **Length of follow-up** | **ID definition** | **Effect (95% CI)** |
| --- | --- | --- | --- | --- | --- | --- |
| Nyakeriga et al 2004 [1] | Kenya | 8 to 96 | 240 | 6 months | Ferritin<12µg/L plus TSAT<10% | 0.70 (0.51, 0.99)^[[5]](#footnote-1)^ |
| Jonker et al 2012 [2] | Malawi | 6 to 60 | 727 | 12 months | Ferritin<30µg/L | 0.49 (0.33, 0.73)^[[6]](#footnote-2)^ |
|  |  |  |  |  | Ferritin<12µg/L if CRP<10mg/L  or <30µg/L if CRP>10mg/L | 0.43 (0.25, 0.73)^b^ |
| Gwamaka et al 2012 [3] | Tanzania | 0 to 36 | 785 | 3 years | Ferritin<30µg/L if CRP<8.2mg/L  or <70µg/L if CRP>8.2mg/L | 0.77 (0.66, 0.89)^[[7]](#footnote-3)^ |
| Barffour et al 2017 [4] | Zambia | 48 to 72 | 745 | 6 months | Ferritin<12µg/L in children<5 years  or <15µg/L in children ≥5 years | 0.63 (0.35, 1.11)^[[8]](#footnote-4)^ |
| Current study | Kenya and Uganda | 0 to 84 | 2504 | 6 months | Ferritin<12µg/L or <30µg/L if CRP>5mg/L in children<5 years or <15µg/L in children ≥5 years | 0.67 (0.56, 0.81)^[[9]](#footnote-5)^ |
|  |  |  |  |  | TSAT<10% | 0.71 (0.54, 0.95)^e^ |

**Supplementary Figure 1. Effect of iron status on malaria risk over a one-year period.** Labels indicate incidence rate ratio and 95% confidence intervals. ID is iron deficiency; IDA, iron deficiency anemia; sTfR, soluble transferrin receptor; TSAT, transferrin saturation. ID-low ferritin was defined as plasma ferritin < 12µg/L or < 30µg/L in the presence of inflammation (CRP > 5mg/L) in children < 5 years or < 15µg/L in children ≥ 5 years otherwise, iron replete. Anemia was defined as hemoglobin < 11g/dL in children aged 0 to 4 years or hemoglobin < 11.5 g/dL in children above 4 years. Iron deficiency anemia was defined as low ferritin and anemia. Poisson regression models were adjusted for age, sex, parasitemia, inflammation, length of follow-up, and study site.

**Supplementary Figure 2. Effect of iron status on malaria risk over a six-month period excluding children with inflammation and parasitemia at baseline.** Labels indicate incidence rate ratio and 95% confidence intervals. Inflammation was defined as C-reactive protein > 5mg/L. ID is iron deficiency; IDA, iron deficiency anemia; sTfR, soluble transferrin receptor; TSAT, transferrin saturation. ID-low ferritin iron deficiency was defined as plasma ferritin < 12 µg/L in children < 5 years or < 15µg/L in children ≥ 5 years otherwise, iron replete. Anemia was defined as hemoglobin < 11g/dL in children aged 0 to 4 years or hemoglobin < 11.5 g/dL in children above 4 years. Iron deficiency anemia was defined as low ferritin and anemia. Poisson regression models were adjusted for age, sex, length of follow-up, and study site. Numbers are 660 in Kenya and 969 in Uganda.

**Supplementary Figure 3. Effect of iron status on malaria risk over a six-month period by parasitemia or inflammation status at baseline**. Par -ve means parasite negative; Par +ve, parasite positive; Inf -ve, inflammation negative and Inf +ve, inflammation positive. Labels indicate incidence rate ratio and 95% confidence intervals. ID is iron deficiency; IDA, iron deficiency anemia; sTfR, soluble transferrin receptor; TSAT, transferrin saturation. ID-low ferritin was defined as plasma ferritin < 12µg/L or < 30µg/L in the presence of inflammation (CRP > 5mg/L) in children < 5 years or < 15µg/L in children ≥ 5 years otherwise, iron replete. Anemia was defined as hemoglobin < 11g/dL in children aged 0 to 4 years or hemoglobin < 11.5 g/dL in children above 4 years. Iron deficiency anemia was defined as low ferritin and anemia. Poisson regression models were adjusted for age, sex, inflammation, parasitemia, length of follow-up, and study site.

**Supplementary Figure 4. Effect of iron deficiency on malaria risk by age groups**. Labels indicate incidence rate ratio and 95% confidence intervals. TSAT, transferrin saturation. ID-low ferritin was defined as plasma ferritin < 12µg/L or < 30µg/L in the presence of inflammation (CRP > 5mg/L) in children < 5 years or < 15µg/L in children ≥ 5 years.

**Supplementary Figure 5. Effect of iron status on malaria risk including adjustment for sickle cell trait.** Labels indicate incidence rate ratio and 95% confidence intervals. ID is iron deficiency; IDA, iron deficiency anemia; sTfR, soluble transferrin receptor; TSAT, transferrin saturation. ID-low ferritin was defined as plasma ferritin < 12µg/L or < 30µg/L in the presence of inflammation (CRP > 5mg/L) in children < 5 years or < 15µg/L in children ≥ 5 years otherwise, iron replete. Anemia was defined as hemoglobin < 11g/dL in children aged 0 to 4 years or hemoglobin < 11.5 g/dL in children above 4 years. Iron deficiency anemia was defined as low ferritin and anemia. Poisson regression models were adjusted for age, sex, parasitemia, inflammation, length of follow-up, sickle cell trait and study site.

**References**

1. Nyakeriga AM, Troye-blomberg M, Chemtai AK, Marsh K, Williams TN. Iron Deficiency and Malaria among Children Living on the Coast of Kenya. J Infect Dis. **2004**;190:439–47.

2. Jonker FAM, Calis JCJ, van Hensbroek MB, Phiri K, Geskus RB, Brabin BJ, et al. Iron status predicts malaria risk in Malawian preschool children. PLoS One. **2012**;7:1–8.

3. Gwamaka M, Kurtis JD, Sorensen BE, Holte S, Morrison R, Mutabingwa TK, et al. Iron deficiency protects against severe plasmodium falciparum malaria and death in young children. Clin Infect Dis. **2012**;54:1137–44.

4. Barffour MA, Schulze KJ, Coles CL, Chileshe J, Kalungwana N, Arguello M, et al. High iron stores in the low malaria season increase malaria risk in the high transmission season in a prospective cohort of rural Zambian children. J Nutr. **2017**;147:1531–6.

1. ID, iron deficiency; HR, Hazard ratio; TSAT, transferrin saturation

   Models were adjusted for age, sex, cohort and inflammation. [↑](#endnote-ref-1)
2. ID (low ferritin) was defined as plasma ferritin < 12µg/L or < 30µg/L in the presence of inflammation (CRP > 5mg/L) in children < 5 years or < 15µg/L in children ≥ 5 years. [↑](#endnote-ref-2)
3. Anemia was defined as hemoglobin < 11g/dL in children aged 0 to 4 years or hemoglobin < 11.5 g/dL in children above 4 years. [↑](#endnote-ref-3)
4. Iron deficiency anemia was defined as low ferritin and anemia. [↑](#endnote-ref-4)
5. Incidence rate ratio of a clinical malaria episode defined as asexual *P. falciparum* parasitemia plus fever/temperature > 37.5^0^C. At baseline, children with inflammation and malaria were excluded. [↑](#footnote-ref-1)
6. Hazards ratio of a clinical malaria episode defined as asexual *P. falciparum* parasitemia plus fever/temperature > 37.5^0^C. [↑](#footnote-ref-2)
7. Indicates odds of asexual *P. falciparum* parasitemia following routine aparasitemic visits. [↑](#footnote-ref-3)
8. Indicates incidence rate ratio of parasitemia recalculated as inverse of what was reported (1.60 (0.90, 2.86)). Ferritin concentrations were corrected for baseline inflammation (measured using alpha 1-acid glycoprotein) and malaria. [↑](#footnote-ref-4)
9. Our current study reports incidence rate ratio of a clinical malaria episode defined as asexual *P. falciparum* parasitemia plus fever/temperature > 37.5^0^C.

   CRP, C-reactive protein; ID, Iron deficiency; TSAT, transferrin saturation. [↑](#footnote-ref-5)
